# Institutional and Regional Variation in Opioid Prescribing for Hospitalized Infants in the US

**DOI:** 10.1001/jamanetworkopen.2024.0555

**Published:** 2024-03-12

**Authors:** Olivia A. Keane, Shadassa Ourshalimian, Ashwini Lakshmanan, Henry C. Lee, Susan R. Hintz, Nam Nguyen, Madeleine C. Ing, Cynthia L. Gong, Cameron Kaplan, Lorraine I. Kelley-Quon

**Affiliations:** 1 Division of Pediatric Surgery, Children’s Hospital Los Angeles, Los Angeles, California; 2 Department of Health Systems Science, Bernard J. Tyson Kaiser Permanente School of Medicine, Pasadena, California; 3 Division of Neonatology, University of California San Diego, La Jolla; 4 Stanford University School of Medicine, Department of Pediatrics, Division of Neonatology, Palo Alto, California; 5 Division of Pediatric Surgery, Memorial Care Miller Children’s & Women’s Hospital, Long Beach, California; 6 Fetal and Neonatal Institute, Division of Neonatology, Department of Pediatrics, Children’s Hospital Los Angeles, Keck School of Medicine, University of Southern California, Los Angeles; 7 USC Gehr Family Center for Health Systems Science and Innovation, Keck School of Medicine, University of Southern California, Los Angeles; 8 Department of Surgery, Keck School of Medicine, University of Southern California, Los Angeles; 9 Department of Population and Public Health Sciences, Keck School of Medicine, University of Southern California, Los Angeles

## Abstract

**Importance:**

High-risk infants, defined as newborns with substantial neonatal-perinatal morbidities, often undergo multiple procedures and require prolonged intubation, resulting in extended opioid exposure that is associated with poor outcomes. Understanding variation in opioid prescribing can inform quality improvement and best-practice initiatives.

**Objective:**

To examine regional and institutional variation in opioid prescribing, including short- and long-acting agents, in high-risk hospitalized infants.

**Design, Setting, and Participants:**

This retrospective cohort study assessed high-risk infants younger than 1 year from January 1, 2016, to December 31, 2022, at 47 children’s hospitals participating in the Pediatric Health Information System (PHIS). The cohort was stratified by US Census region (Northeast, South, Midwest, and West). Variation in cumulative days of opioid exposure and methadone treatment was examined among institutions using a hierarchical generalized linear model. High-risk infants were identified by *International Statistical Classification of Diseases and Related Health Problems, Tenth Revision* codes for congenital heart disease surgery, medical and surgical necrotizing enterocolitis, extremely low birth weight, very low birth weight, hypoxemic ischemic encephalopathy, extracorporeal membrane oxygenation, and other abdominal surgery. Infants with neonatal opioid withdrawal syndrome, in utero substance exposure, or malignant tumors were excluded.

**Exposure:**

Any opioid exposure and methadone treatment.

**Main Outcomes and Measures:**

Regional and institutional variations in opioid exposure.

**Results:**

Overall, 132 658 high-risk infants were identified (median [IQR] gestational age, 34 [28-38] weeks; 54.5% male). Prematurity occurred in 30.3%, and 55.3% underwent surgery. During hospitalization, 76.5% of high-risk infants were exposed to opioids and 7.9% received methadone. Median (IQR) length of any opioid exposure was 5 (2-12) cumulative days, and median (IQR) length of methadone treatment was 19 (7-46) cumulative days. There was significant hospital-level variation in opioid and methadone exposure and cumulative days of exposure within each US region. The computed intraclass correlation coefficient estimated that 16% of the variability in overall opioid prescribing and 20% of the variability in methadone treatment was attributed to the individual hospital.

**Conclusions and Relevance:**

In this retrospective cohort study of high-risk hospitalized infants, institution-level variation in overall opioid exposure and methadone treatment persisted across the US. These findings highlight the need for standardization of opioid prescribing in this vulnerable population.

## Key Points

**Question:**

How does the prescribing of short-acting opioids and methadone in high-risk infants vary across institutions and regions of the US?

**Findings:**

In this cohort study of 132 658 high-risk infants, there was significant hospital-level variation in opioid and methadone exposure and cumulative days received. The study estimated that 16% of the variability in any opioid exposure and 20% of the variability in methadone treatment was attributable to the individual hospital.

**Meaning:**

These findings suggest that institution-level variation in opioid and methadone prescribing in hospitalized infants persists across US children’s hospitals, underscoring the need for standardized prescribing in this vulnerable population.

## Teaser

This cohort study examines regional and institutional variation in overall opioid exposure and methadone treatment in high-risk infants at US children’s hospitals.

## Introduction

High-risk infants, defined as newborns with neonatal-perinatal morbidities, often receive pain control for procedures and prolonged intubation.^1,2,3^ Infants exposed to painful procedures experience acute physiologic responses and increased morbidity, and opioids reduce these poor outcomes.^4,5^ However, extended opioid prescribing after surgery is associated with prolonged ventilation, total parenteral nutrition use, and hospitalization.^1,6,7,8,9^ Furthermore, higher cumulative opioid exposure is associated with impaired neurodevelopment, including impaired cerebellar growth, developmental disability, and poor socialization.^10,11,12,13,14^ Ultimately, regional and hospital-level differences in hospitalized infants’ opioid exposure may have significant effects on both short- and long-term clinical outcomes.

Regional opioid prescribing differences in the US are well described in adults.^15^ Most pediatric literature focuses on single-institution opioid prescribing and outpatient prescribing.^16,17,18,19,20^ Infants infrequently receive outpatient opioids, with most opioid exposure occurring during hospitalization.^21^ Few studies examine institutional variation in inpatient opioid prescribing for critically ill infants and children.^1,2,3,9^ In addition to potentially worse clinical outcomes, high-risk infants in centers that more commonly prescribe opioids likely have increased short-term costs and increased long-term health care use.^21,22,23,24,25,26^ Understanding regional and institutional variation can inform best-practice initiatives and encourage practitioners to carefully consider when opioids are the appropriate medications for the patient.

This study examined regional and institutional variation in overall opioid exposure and methadone treatment in high-risk infants cared for at children’s hospitals in the US. We hypothesized that significant variation exists across US regions and among institutions in opioid exposure and methadone treatment among high-risk hospitalized infants.

## Methods

### Study Design

A cohort of high-risk infants younger than 1 year at the time of admission from January 1, 2016, to December 31, 2022, was identified using the Pediatric Health Information Systems (PHIS) database. The PHIS database is maintained by the Children’s Hospital Association and includes clinical and resource utilization data for both inpatient and outpatient encounters for more than 47 children’s hospitals across the US. Hospitals registered in the PHIS database include freestanding children’s hospitals and large pediatric hospitals within an academic health system.^27^ All data are deidentified, and data integrity is checked by the Children’s Hospital Association data quality program, which issues quarterly reports to participating hospitals that detail any quality concerns. The Children's Hospital Los Angeles Institutional Review Board approved the study and waived the need for informed consent given the use of deidentified data. This study followed the Strengthening the Reporting of Observational Studies in Epidemiology (STROBE) reporting guideline.

High-risk infants are newborns with significant perinatal-neonatal morbidities.^1,2,3^ We defined high-risk infants using *International Statistical Classification of Diseases and Related Health Problems, Tenth Revision* (*ICD-10*) codes (eTable 1 in Supplement 1) that included diagnoses in the following categories: congenital heart disease (CHD) procedure, necrotizing enterocolitis (NEC), extremely low birth weight (ELBW), very low birth weight (VLBW), hypoxemic ischemic encephalopathy (HIE), extracorporeal membrane oxygenation (ECMO), and other abdominal surgery. These categories were defined by *ICD-10* codes for each patient encounter and thus are not mutually exclusive. Each patient may fall into multiple categories.

Patients with the following *ICD-10* codes were excluded: P96.1 (neonatal withdrawal symptoms from maternal use of drugs of addiction), P96.2 (withdrawal symptoms from therapeutic use of drugs in newborn), and P90.49 (newborn affected by maternal use of other drugs of addiction) (n = 7055) (eFigure in Supplement 1). Patients receiving methadone alone and no other opioid exposure were excluded because receipt of methadone alone likely captured infants with in utero opioid exposure undergoing treatment for withdrawal prevention (n = 110). Additionally, patients with malignant tumors as defined by Feudtner et al^28^ were excluded because pain management in these patients is complex and can be chronic and/or palliative in nature (n = 1895). Infants who did not undergo a cardiac or abdominal surgical intervention and had a CHD or abdominal surgical diagnosis code alone were excluded (cardiac: n = 139 647, abdominal: n = 35 495) (eFigure in Supplement 1).

The cohort was divided into US Census and PHIS regions based on hospital location. The regional distribution of the 47 institutions was as follows: Northeast, 6; South, 17; Midwest, 14; and West, 10. Individual hospitals were deidentified so that variation among institutions could be examined. Demographic and clinical factors were characterized and included the following: gestational age at admission (weeks), biologic sex, birth weight (kilograms), race, ethnicity, insurance type, mechanical ventilation, intensive care unit (ICU) stay, and number of complex chronic conditions. Race and ethnicity were included in our study because race and ethnicity have been shown to influence prescribing decisions in pediatric populations.^29,30,31^ Race categories were defined according to the PHIS and included American Indian, Asian, Black, Pacific Islander, White, other (a defined race category in the PHIS database), and unknown.^32^ Similarly, ethnicity categories (Hispanic, non-Hispanic, and unknown) were determined via the PHIS.^32^ Hospitals submit race and ethnicity data to the PHIS for each visit according to hospital-specific practices, which may include parent or guardian report at the time of hospital registration. Additionally, the literature suggests that sex-based differences in behavior and gene expression are associated with neonatal opioid exposure.^33,34^ Insurance or payer type is also associated with opioid prescribing both postoperatively and in hospitalized pediatric patients.^29,35,36^

### Outcome Measure

The primary outcome of interest was any opioid exposure (including short-acting opioids and methadone) during hospitalization. Opioids were identified based on pharmacy billing codes for opioid analgesic. Additional outcomes of interest included cumulative days of opioid exposure, methadone treatment after short-acting opioid exposure during hospitalization, and cumulative methadone days.

### Statistical Analysis

Continuous variables were described using median (IQR), and Wilcoxon-Mann-Whitney tests were used to compare findings among different regions. Categorical variables were described using numbers (percentages), and χ^2^ or Fisher exact tests were used to compare findings among groups. The Kruskal-Wallis test was used to examine the difference in distributions between the cumulative days of exposure to opioids and methadone.

A 2-level hierarchical generalized linear model (HGLM) with hospitals as the random intercepts was used to evaluate variation in opioid prescribing; patients (at level 1) were nested within institutions (at level 2).^37^ Institution-level variation in any opioid prescribing was estimated by computing the intraclass correlation coefficient, which indicates how much of the total variation in the probability of use during a patient encounter is attributed to the hospital. Due to the nonnormal distribution of data, an HGLM model was executed to examine the effects of demographic and clinical factors on natural log-transformed cumulative days of opioid exposure during hospitalization. Results were then back-transformed for ease of interpretation. Because most of the cohort was composed of infants with CHD requiring intervention, these infants were used as the reference group. Covariates (sex, race, ethnicity, insurance type, prematurity, high-risk category, mechanical ventilatory support, ICU stay, and complex chronic conditions) were chosen a priori,^14,29,30,31,32,33,34,35,36^ on the basis of clinical expertise, and from significant bivariate associations. Model fit was checked via the Akaike information criterion and the Bayes information criterion, where lower values indicate improved fit.^38^ All analyses were conducted with an α = .05. Data were analyzed using SAS software, version 9.4 (SAS Institute Inc).

## Results

Following exclusions, a cohort of 132 658 high-risk infants was evaluated (0.4% American Indian, 3.3% Asian, 20.9% Black, 0.7% Pacific Islander, 52.2% White, 15.4% other, and 7.1% unknown; 17.8% Hispanic, 73.4% non-Hispanic, and 8.7% unknown ethnicity; 54.5% male, 45.4% female, and 0.1% unknown sex; and 54.7% with public insurance) (Table 1). The median (IQR) gestational age was 34 (28-38) weeks, and median (IQR) birth weight was 1741 (1077-3055) g. The most common high-risk diagnosis was CHD (65.5%), prematurity occurred in 30.3% of infants, and 55.3% underwent a procedural intervention. Infants with ELBW had a median (IQR) length of stay of 76 (17-119) days, and infants with VLBW had a median (IQR) length of stay of 36 (19-62) days. During hospitalization, 52.6% of the high-risk infant cohort required mechanical ventilation for a reason apart from procedural intervention. There were significant regional differences in demographic and clinical factors, including high-risk diagnosis category, proportion of patients undergoing surgical intervention, and number of high-risk procedures. The Northeast and West regions had a higher percentage of patients who were surgically treated during the index hospitalization.

**Table 1.** Demographic and Clinical Characteristics of the Total Cohort and by US Region^a^

Characteristic	Total (N = 132 658)	Midwest (n = 37 627)	Northeast (n = 18 037)	South (n = 51 240)	West (n = 25 574)	*P* value
Length of stay, median (IQR), d	23 (8-59)	26 (9-66)	20 (8-54)	23 (8-62)	19 (7-49)	<.001
Gestational age, median (IQR), wk	34 (28-38)	33 (28-38)	35 (29-38)	34 (28-38)	35 (29-39)	<.001
Birth weight, median (IQR), g	1741 (1077-3055)	1680 (1070-2975)	2170 (1140-3130)	1740 (1020-3020)	2225.5 (1185-3170)	<.001
Sex						
Male	72 270 (54.5)	20 276 (53.9)	10 094 (56.0)	27 699 (53.9)	14 201 (55.5)	<.001
Female	60 289 (45.4)	17 333 (46.1)	7927 (43.9)	23 677 (46.0)	11 352 (44.4)	
Unknown	99 (0.1)	18 (0)	16 (0.1)	44 (0.1)	21 (0.1)	
Race						
American Indian	541 (0.4)	163 (0.4)	53 (0.3)	127 (0.2)	198 (0.8)	<.001
Asian	4333 (3.3)	1071 (2.8)	519 (2.9)	1216 (2.4)	1527 (6.0)	
Black	27 776 (20.9)	8440 (22.4)	2735 (15.2)	15 312 (29.8)	1289 (5.0)	
Pacific Islander	917 (0.7)	105 (0.3)	24 (0.1)	152 (0.3)	636 (2.5)	
White	69 244 (52.2)	22 060 (58.6)	7654 (42.4)	26 327 (51.2)	13 203 (51.6)	
Other^b^	20 378 (15.4)	2989 (7.9)	4063 (22.5)	6155 (12.0)	7171 (28.0)	
Unknown	9469 (7.1)	2799 (7.4)	2989 (16.6)	2131 (4.1)	1550 (6.1)	
Ethnicity						
Hispanic	23 677 (17.8)	2413 (6.4)	2211 (12.3)	9407 (18.3)	9646 (37.7)	<.001
Non-Hispanic	97 422 (73.4)	32 685 (86.9)	11 838 (65.6)	38 999 (75.8)	13 900 (54.4)	
Unknown	11 559 (8.7)	2529 (6.7)	3988 (22.1)	3014 (5.9)	2028 (7.9)	
Insurance						
Private	53 048 (40.0)	15 677 (41.7)	8580 (47.6)	18 357 (35.7)	10 434 (40.8)	<.001
Public	72 543 (54.7)	20 924 (55.6)	8577 (47.6)	31 470 (61.2)	11 572 (45.2)	
Other	7067 (5.3)	1026 (2.7)	880 (4.9)	1593 (3.1)	3568 (14)	
Prematurity	40 174 (30.3)	13 224 (35.1)	4429 (24.6)	16 093 (31.3)	6428 (25.1)	<.001
High-risk diagnosis						
CHD diagnosis	86 832 (65.5)	22 526 (59.9)	12 288 (68.1)	34 292 (66.7)	17 726 (69.3)	<.001
CHD surgery	51 581 (38.9)	12110 (32.2)	8509 (47.2)	19 738 (38.4)	11 224 (43.9)	<.001
Abdominal surgery	20 927 (15.8)	5700 (15.1)	2781 (15.4)	8256 (16.1)	4190 (16.4)	<.001
Medical NEC	9194 (6.9)	2678 (7.1)	1111 (6.2)	3955 (7.7)	1450 (5.7)	<.001
Surgical NEC	3111 (2.3)	791 (2.1)	429 (2.4)	1358 (2.6)	533 (2.1)	<.001
VLBW	25 413 (19.2)	9210 (24.5)	2937 (16.3)	9116 (17.7)	4150 (16.2)	<.001
ELBW	24 397 (18.4)	7771 (20.7)	2809 (15.6)	10 022 (19.5)	3795 (14.8)	<.001
HIE	14 115 (10.6)	3792 (10.1)	1436 (8)	5649 (11)	3238 (12.7)	<.001
ECMO	6062 (4.6)	1626 (4.3)	997 (5.5)	2354 (4.6)	1085 (4.2)	<.001
No. of diagnoses						
0	8749 (6.6)	2400 (6.4)	1267 (7)	3331 (6.5)	1751 (6.8)	<.001
1	87 286 (65.8)	24 499 (65.1)	12 633 (70)	33 029 (64.2)	17 125 (67.0)	
2	32 394 (24.4)	9488 (25.2)	3701 (20.5)	13 196 (25.7)	6009 (23.5)	
≥3	4229 (3.2)	1240 (3.3)	436 (2.4)	1864 (3.6)	689 (2.7)	
Surgery (yes/no)	73 294 (55.3)	18 071 (48.0)	11 436 (63.4)	28358 (55.1)	15 429 (60.3)	<.001
No. of high-risk procedures						
0	59 364 (44.7)	19 556 (52)	6601 (36.6)	23 062 (44.9)	10 145 (39.7)	<.001
1	67 140 (50.6)	16 496 (43.8)	10 552 (58.5)	25 851 (50.3)	14 241 (55.7)	
2	5605 (4.2)	1427 (3.8)	816 (4.5)	2275 (4.4)	1087 (4.3)	
≥3	549 (0.4)	148 (0.4)	68 (0.4)	232 (0.5)	101 (0.4)	
Mechanical ventilatory support (yes/no)	69 736 (52.6)	18 778 (49.9)	10 107 (56.0)	28 486 (55.4)	12 365 (48.3)	<.001
Hospital unit						
NICU	76 960 (58.0)	22 962 (61.0)	9849 (54.6)	30 420 (59.2)	13 729 (53.7)	<.001
ICU	52 253 (39.4)	14 026 (37.3)	7852 (43.5)	19 687 (38.3)	10 688 (41.8)	<.001

Abbreviations: CHD, congenital heart disease; ECMO, extracorporeal membrane oxygenation; ELBW, extremely low birth weight; HIE, hypoxemic ischemic encephalopathy; ICU, intensive care unit; NEC, necrotizing enterocolitis; NICU, neonatal intensive care unit; VLBW, very low birth weight.

^a^ Data are presented as number (percentage) of patients unless otherwise indicated.

^b^ “Other” is a defined race category in the Pediatric Health Information Systems database.

During hospitalization, 76.5% of high-risk infants were prescribed opioids. When examining specific opioid type during hospitalization, 66.5% of high-risk infants were exposed to fentanyl, 60.6% to morphine, 5.8% to hydromorphone, and 7.9% to methadone (Table 2). On bivariate comparison, opioid and methadone exposure significantly varied by US region (Table 2). Examining any opioid prescribing within regions showed that of high-risk infants, 74.2% in the Northeast, 78.6% in the South, 71.6% in the Midwest, and 81.2% in the West were exposed to opioids. Similarly, of high-risk infants, 4.6% in the Northeast, 10.3% in the South, 6.6% in the Midwest, and 7.1% in the West were exposed to methadone. Notably, when infants were stratified by preterm vs term birth, we found that premature infants had less exposure to opioids and methadone than term infants (eTable 2 in Supplement 1).

**Table 2.** Opioid and Methadone Exposures in Total Cohort and US Region

Exposure (yes/no)	No. (%) of infants					*P* value
	Total cohort (N = 132 658)	Midwest (n = 37 627)	Northeast (n = 18 037)	South (n = 51 240)	West (n = 25 574)	
Any opioids, including methadone	101 471 (76.5)	26 936 (71.6)	13 385 (74.2)	40 396 (78.6)	20 754 (81.2)	<.001
Opioids, excluding methadone	101 471 (76.5)	26 936 (71.6)	13 385 (74.2)	40 396 (78.6)	20 754 (81.2)	<.001
Fentanyl	88 253 (66.5)	23 473 (62.4)	10 812 (59.9)	36 255 (70.5)	17 713 (69.3)	<.001
Morphine	80 454 (60.6)	21 019 (55.9)	11 083 (61.4)	30 761 (59.8)	17 591 (68.8)	<.001
Hydromorphone	7725 (5.8)	1655 (4.4)	814 (4.5)	2816 (5.5)	2440 (9.5)	<.001
Methadone	10 426 (7.9)	2494 (6.6)	837 (4.6)	5278 (10.3)	1817 (7.1)	<.001

Across the total cohort, the median (IQR) duration of any opioid exposure was 5 (2-12) cumulative days, and the median (IQR) duration of methadone exposure was 19 (7-46) cumulative days. Despite median days of opioid exposure between US regions being equal (5 days), cumulative days of opioid use significantly varied by US region due to differences in data distribution and IQR among regions (eTable 3 in Supplement 1). Median (IQR) cumulative days of methadone exposure was 23 (11-54) days in the Northeast, 18 (7-43) days in the South, 21 (7-53) days in the Midwest, and 16 (6-43) days in the West (eTable 3 in Supplement 1). When groups were stratified by prematurity, premature infants had fewer cumulative days of opioid exposure than term infants (eTable 4 in Supplement 1).

In the HGLM duration multivariable regression model evaluating cumulative days of any opioid exposure, the demographic factors found to be associated with decreased days of any opioid exposure included female compared with male sex (0.94; 95% CI, 0.93-0.95), Asian compared with White race (0.94; 95% CI, 0.91-0.98), and private vs public insurance (0.96; 95% CI, 0.94-0.97) (Table 3). Although the Northeast region was associated with decreased days of opioid exposure when compared with the West (0.82; 95% CI, 0.70-0.97), no other US region was significantly associated with days of opioid exposure. Notably, cumulative days of opioid exposure in premature infants was 13% lower (95% CI, 16% to 11%) lower compared with infants who were full term. Other clinical factors associated with higher cumulative opioid days included mechanical ventilation compared with no mechanical ventilation (2.17; 95% CI, 2.14-2.20) and ICU stay compared with hospital bed (1.67; 95% CI, 1.63-1.72). A higher percentage of ELBW infants received ventilatory support when compared with VLBW infants (69.3% vs 37.6%). Additionally, cumulative days of opioid exposure increased for each 1-unit increase in the number of complex chronic conditions (1.36; 95% CI, 1.36-1.37) (Table 3). Additionally, all but 1 (VLBW) of the high-risk infant diagnosis categories were associated with increased cumulative opioid days. Cumulative opioid days in patients with surgical NEC were 104% (95% CI, 99%-112%) higher compared with patients without surgical NEC. Similarly, cumulative opioid days were 52% (95% CI, 49%-56%) higher in infants with medical NEC, 82% (95% CI, 78%-86%) higher in those who underwent abdominal surgery, 25% (95% CI, 21%-28%) higher in those with ELBW, 105% (95% CI, 102%-109%) higher in those who had a CHD-related procedure, and 177% (95% CI, 169%-185%) higher in those receiving ECMO (Table 3).

**Table 3.** Association of Demographic and Clinical Characteristics With Cumulative Days of Opioid Exposure

Characteristic	Estimate (95% CI)^a^	*P* value
Sex		
Male	1.0 [Reference]	NA
Female	0.94 (0.93-0.95)	<.001
Race		
Asian	0.94 (0.91-0.98)	.001
Black	0.99 (0.97-1.01)	.22
White	1.0 [Reference]	NA
Other^b^	0.99 (0.97-1.01)	.34
Ethnicity		
Hispanic	1.01 (0.97-1.04)	.60
Not Hispanic	1.01 (0.98-1.04)	.60
Unknown	1.0 [Reference]	NA
Insurance		
Private	0.96 (0.94-0.97)	<.001
Public	1.0 [Reference]	NA
Other	0.91 (0.88-0.94)	<.001
US region		
Midwest	0.96 (0.84-1.11)	.59
Northeast	0.82 (0.70-0.97)	.02
South	0.92 (0.81-1.06)	.24
West	1.0 [Reference]	NA
Prematurity		
No	1.0 [Reference]	NA
Yes	0.87 (0.84-0.89)	<.001
High-risk infant category		
CHD diagnosis	2.05 (2.02-2.09)	<.001
Abdominal surgery	1.82 (1.78-1.86)	<.001
Medical NEC	1.52 (1.49-1.56)	<.001
Surgical NEC	2.04 (1.97-2.12)	<.001
VLBW	0.90 (0.87-0.93)	<.001
ELBW	1.25 (1.21-1.28)	<.001
HIE	1.07 (1.05-1.10)	<.001
ECMO	2.77 (2.69-2.85)	<.001
Mechanical ventilation	2.17 (2.14-2.20)	<.001
ICU stay		
No	1.0 [Reference]	NA
Yes	1.67 (1.63-1.72)	<.001
No. of complex chronic conditions (continuous)	1.36 (1.36-1.37)	<.001

Abbreviations: CHD, congenital heart disease; ECMO, extracorporeal membrane oxygenation; ELBW, extremely low birth weight; HIE, hypoxemic ischemic encephalopathy; ICU, intensive care unit; NA, not applicable; NEC, necrotizing enterocolitis; VLBW, very low birth weight.

^a^ Reference diagnosis of congenital heart disease. Outcome was back-transformed.

^b^ “Other” is a defined race category in the Pediatric Health Information Systems database.

**Figure 1.**
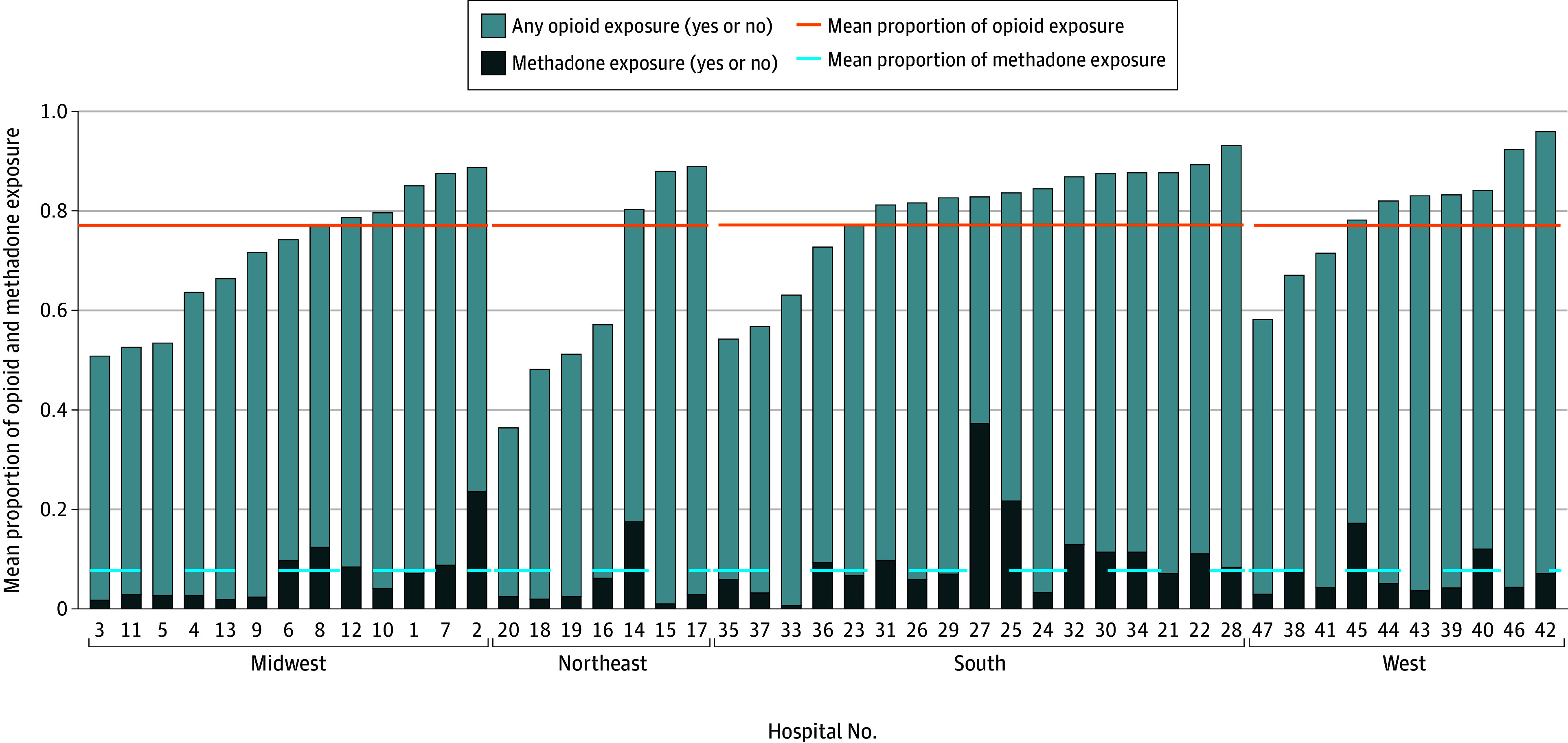
Institutional Variation in Any Opioid and Methadone Exposure by US Region Individual institutions were deidentified and assigned a study identification number.

**Figure 2.**
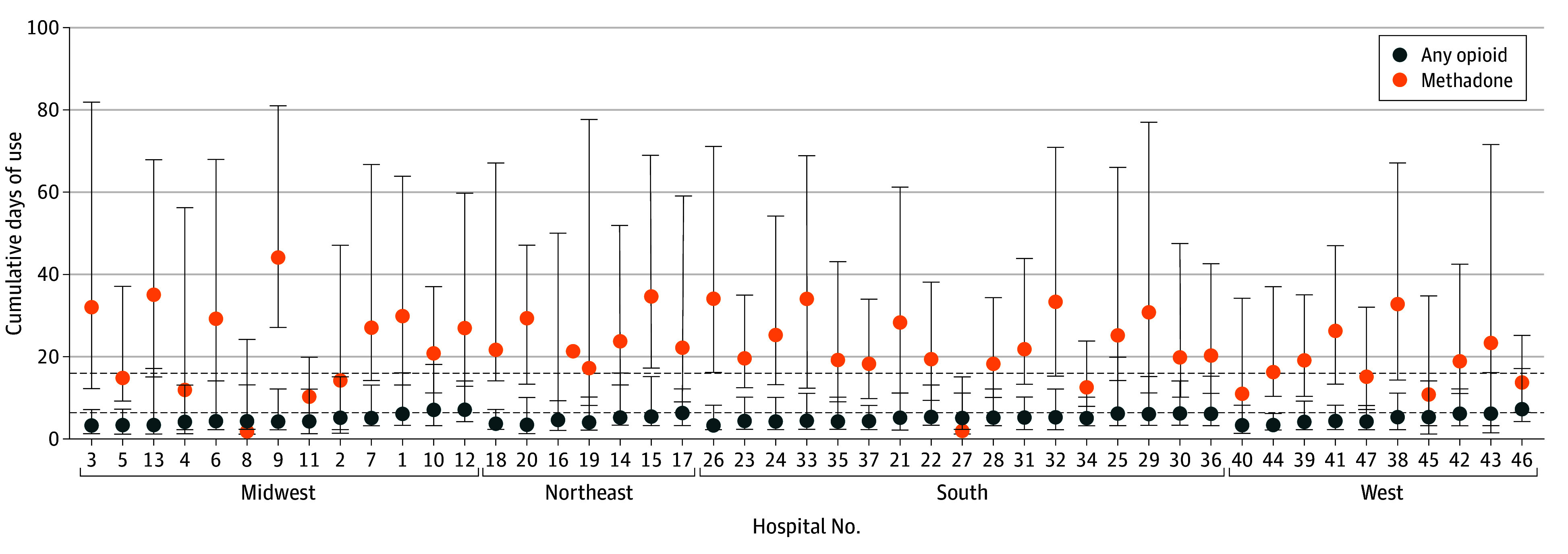
Institutional Variation in Cumulative Days of Any Opioid and Methadone Exposure by US Region Individual institutions were deidentified and assigned a study identification number. Data points indicate medians; error bars, IQRs.

Significant hospital-level variation in opioid and methadone exposure was demonstrated within each region (Figure 1). Similar wide variation in cumulative days of opioid and methadone exposure was seen across institutions within each US region (Figure 2). Results from our HGLM unconditional model were used to calculate the intraclass correlation coefficient. The probability of opioid exposure at a typical US children’s hospital was 78%, but the probability of opioid exposure varied considerably across institutions. The probability of methadone exposure after short-acting opioid exposure in high-risk infants at a typical US children’s hospital was 6%, but the probability of methadone exposure similarly varied considerably across hospitals. Approximately 16% of the variability in opioid prescribing and 20% of the variability in methadone treatment were attributable to the individual hospital. Therefore, 84% of the variability in opioid prescribing and 80% of the variability in methadone treatment were accounted for by individual patient-level characteristics and possible unobserved or unknown characteristics.

## Discussion

In this retrospective cohort study of 132 658 high-risk infants admitted to 47 children’s hospitals in the US, we found that most received opioids during hospitalization with wide variation across US regions and between hospitals. Furthermore, 16% of the variability in any opioid prescribing and 20% of the variability in methadone treatment was attributable to the individual hospital. This is the first study, to our knowledge, to describe regional variation and quantify institutional variation in inpatient opioid exposure and duration of prescribing in high-risk infants. Our findings underscore the magnitude of variation at children’s hospitals across the US, which likely has significant implications for short- and long-term clinical outcomes and resource use.

Adult data indicate that state and regional patterns in prescribing have remained relatively consistent, with areas of high prescribing persisting despite national efforts to decrease opioid exposure.^15^ Similar findings have been reported in the pediatric literature examining outpatient prescription opioids.^18^ Although institutional variation in inpatient opioid prescribing has been described in certain pediatric populations, there is limited literature examining regional variation in opioid exposure during hospitalization.^39,40,41,42^ Our study found that once hospital- and patient-level characteristics were controlled for, regional variation was no longer significantly associated with opioid prescribing, with the exception of the Northeast region. These findings imply that local and institutional quality improvement efforts are needed to minimize variation in infant opioid prescribing.

Notably, opioids can decrease morbidity that can result from the physiologic response to pain in infants.^4,5^ However, prolonged exposure in neonatal surgical populations is associated with increased health care use, including prolonged hospitalization, total parenteral nutrition use, and mechanical ventilation.^9^ Additionally, prolonged opioid exposure can lead to impaired neurodevelopment.^6,7,8,11^ Viewed collectively, our results underscore the need for individual hospitals to track cumulative opioid exposures and methadone use for hospitalized infants to improve clinical and possibly neurodevelopmental outcomes.

Our study aligns with previous literature demonstrating wide institutional variation in inpatient opioid prescribing in the US and Canada for critically ill infants and children.^1,2,3,9,39^ Importantly, despite the significant institutional variation in opioid prescribing, 84% of the variability in opioid exposure and 80% of the variability in methadone treatment were accounted for by individual patients and other unknown characteristics. Genetic variants in opioid receptors and stress response genes have been found to be associated with differences in infant pharmacologic treatment responses and length of hospitalization.^43,44,45,46^ Investigations into genetic predisposition to poor opioid response have been conducted in infants receiving mechanical ventilation. A study by Elens et al^47^ concluded that specific genetic alleles predispose premature infants to diminished opioid-induced pain relief. Further investigation of genetic factors that influence opioid response in high-risk infants could explain the variability in opioid prescribing attributed to unknown factors.

In the hierarchical analysis, patients requiring mechanical ventilation demonstrated higher cumulative opioid days of exposure. Use of opioids as sedation in infants receiving mechanical ventilation has been increasing over time despite illness severity not increasing.^48^ Our findings highlight the need for sedation protocols for high-risk infants receiving mechanical ventilatory support. Furthermore, our findings support that female sex, Asian race, and private insurance status are associated with fewer cumulative opioid days. Race, ethnicity, and insurance status were included in our models because these demographic factors may influence opioid-prescribing patterns in pediatric populations.^29,30,31,35,36^ Additionally, emerging preclinical data suggest that there are sex-based differences in behavior and gene expression associated with neonatal opioid exposures and metabolism.^33,34^ Importantly, our findings highlight that specific subpopulations within high-risk infants are expected to have more cumulative days of receiving opioids, including those with surgical NEC, ECMO, CHD, and ELBW and those undergoing abdominal surgery. Infants with surgical NEC are at risk for increased opioid exposure and methadone use,^9^ as well as inadequate pain control despite opioid administration.^49^ Similarly, initiation of ECMO is associated with substantial sedative exposure and increased frequency of iatrogenic withdrawal syndrome.^50,51^ Furthermore, infants with CHD are at increased risk for neurodevelopmental disorders and deficits, which may make them especially vulnerable in the setting of increased cumulative opioid days of exposure reported in our study.^52,53,54^

Interestingly, although prematurity and VLBW were both associated with decreased cumulative opioid days, ELBW was associated with increased opioid days. Previous studies examining the effects of opioids on premature infants support judicious use of opioids and may have influenced current practices.^55,56^ However, ELBW infants often require prolonged hospitalization and mechanical ventilation; thus, their clinical need for opioids could be greater. In our cohort, ELBW infants had a median (IQR) length of stay of 76 (17-119) days, and VLBW infants had a median (IQR) length of stay of 36 (19-62) days. Additionally, a higher percentage of ELBW infants received ventilation when compared with VLBW infants (69.3% vs 37.6%), which likely explains the differential effect on cumulative opioid days. Notably, a higher cumulative opioid dose is associated with worse cognitive outcomes at 20 months in ELBW infants.^11^ Our findings highlight that certain infant populations are at increased risk for prolonged opioid exposure and thus would likely benefit the most from standardized opioid protocols.

Expanded efforts to quantify and report opioid prescribing for children have led to the development of evidence-based prescribing guidelines and quality improvement initiatives.^57,58,59,60,61,62,63^ However, these efforts largely exclude infants, despite opioid exposure having unique neurodevelopmental risks in this age group. Future studies are needed to examine the long-term effects of prolonged opioid exposures in high-risk infants and further understand the impact of variations in institutional prescribing. Ultimately, opioid stewardship efforts encouraging judicious opioid prescribing should also extend to infants.

### Limitations

This study has some limitations. Of note, use of an administrative database (PHIS) leads to a nonrandom sample of hospitals, which, although large in number, may have different opioid prescribing practices than hospitals not included in the PHIS. The PHIS database contains data on patients cared for at children’s hospitals, which limits generalizability because this may not be representative of high-risk infants admitted to other types of hospitals. Additionally, the PHIS does not include medication dosage or frequency. Thus, it is possible that the observed hospital-level variation may reflect differences in institutional prescribing trends. For example, an institution that administers opioids daily could be administering fewer total morphine equivalents than a center that uses larger doses but for fewer days. Furthermore, there is little context for the variation observed, and conjecture is needed to deduce prescribing choices. Similarly, clinician variability and clinician-level characteristics and practices are not examined within this study and may have contributed to the observed institutional variation. The use of multimodal pain management strategies, including nonopioid pain medications, regional anesthetics, and nonpharmacologic interventions, was also not examined and may have influenced variation.

## Conclusions

Institution-level variation in overall opioid prescribing and methadone treatment in high-risk hospitalized infants persists across US children’s hospitals. These findings highlight a need to develop standardized evidence-based protocols to manage procedural pain, prolonged intubation, and surgical recovery for high-risk infants. Clinical subgroups, including those with surgical NEC, ECMO, CHD, and ELBW and those undergoing abdominal surgery, have increased risk of prolonged opioid exposure and may benefit the most from standardization.

## Notes

Supplement 1. **eTable 1.** ICD-10 Codes Used to Build Study Cohort **eTable 2.** Opioid and Methadone Exposure in Premature vs Term Infants and Stratified by U.S. Region **eTable 3.** Cumulative Days of Opioid and Methadone Exposure in Study Cohort and Stratified by U.S. Region **eTable 4.** Cumulative Days of Opioid and Methadone Exposure in Premature vs Term Infants and Stratified by U.S. Region **eFigure.** Study Flow Diagram

Supplement 2.
Data Sharing Statement

## References

[1] Dervan LA, Yaghmai B, Watson RS, Wolf FM. The use of methadone to facilitate opioid weaning in pediatric critical care patients: a systematic review of the literature and meta-analysis. Paediatr Anaesth. 2017;27(3):228-239. doi:10.1111/pan.13056 28109052

[2] Womer J, Zhong W, Kraemer FW, . Variation of opioid use in pediatric inpatients across hospitals in the U.S. J Pain Symptom Manage. 2014;48(5):903-914. doi:10.1016/j.jpainsymman.2013.12.241 24703942

[3] Borenstein-Levin L, Synnes A, Grunau RE, Miller SP, Yoon EW, Shah PS; Canadian Neonatal Network Investigators. Narcotics and sedative use in preterm neonates. J Pediatr. 2017;180:92-98.e1. doi:10.1016/j.jpeds.2016.08.031 27614931

[4] Anand KJ, Hickey PR. Pain and its effects in the human neonate and fetus. N Engl J Med. 1987;317(21):1321-1329. doi:10.1056/NEJM198711193172105 3317037

[5] Anand KJ, Barton BA, McIntosh N, . Analgesia and sedation in preterm neonates who require ventilatory support: results from the NOPAIN trial. Neonatal Outcome and Prolonged Analgesia in Neonates. Arch Pediatr Adolesc Med. 1999;153(4):331-338. doi:10.1001/archpedi.153.4.331 10201714

[6] Menon G, McIntosh N. How should we manage pain in ventilated neonates? Neonatology. 2008;93(4):316-323. doi:10.1159/000121458 18525216

[7] Kelley-Quon LI, Zamora AK, Ourshalimian S, . Iatrogenic opioid withdrawal in hospitalized infants. J Perinatol. 2022;42(3):399-400. doi:10.1038/s41372-022-01332-6 35169229 PMC8995048

[8] Lewis T, Erfe BL, Ezell T, Gauda E. Pharmacoepidemiology of opiate use in the neonatal ICU: Increasing cumulative doses and iatrogenic opiate withdrawal. J Opioid Manag. 2015;11(4):305-312. doi:10.5055/jom.2015.0279 26312957 PMC4652640

[9] Keane OA, Zamora AK, Ourshalimian S, . Opioid and methadone use for infants with surgically treated necrotizing enterocolitis. JAMA Netw Open. 2023;6(6):e2318910. doi:10.1001/jamanetworkopen.2023.18910 37347485 PMC10288332

[10] Zwicker JG, Miller SP, Grunau RE, . Smaller cerebellar growth and poorer neurodevelopmental outcomes in very preterm infants exposed to neonatal morphine. J Pediatr. 2016;172:81-87.e2. doi:10.1016/j.jpeds.2015.12.024 26763312 PMC5462546

[11] Kocek M, Wilcox R, Crank C, Patra K. Evaluation of the relationship between opioid exposure in extremely low birth weight infants in the neonatal intensive care unit and neurodevelopmental outcome at 2 years. Early Hum Dev. 2016;92:29-32. doi:10.1016/j.earlhumdev.2015.11.001 26624803

[12] Anand KJ, Anderson BJ, Holford NH, ; NEOPAIN Trial Investigators Group. Morphine pharmacokinetics and pharmacodynamics in preterm and term neonates: secondary results from the NEOPAIN trial. Br J Anaesth. 2008;101(5):680-689. doi:10.1093/bja/aen248 18723857 PMC2733178

[13] Ferguson SA, Ward WL, Paule MG, Hall RW, Anand KJ. A pilot study of preemptive morphine analgesia in preterm neonates: effects on head circumference, social behavior, and response latencies in early childhood. Neurotoxicol Teratol. 2012;34(1):47-55. doi:10.1016/j.ntt.2011.10.008 22094261

[14] Puia-Dumitrescu M, Comstock BA, Li S, ; PENUT Consortium. Assessment of 2-year neurodevelopmental outcomes in extremely preterm infants receiving opioids and benzodiazepines. JAMA Netw Open. 2021;4(7):e2115998. doi:10.1001/jamanetworkopen.2021.15998 34232302 PMC8264640

[15] Centers for Disease Control and Prevention. US opioid prescribing maps. Accessed May 31, 2023. https://www.cdc.gov/drugoverdose/rxrate-maps/index.html

[16] Hunsberger JB, Monitto CL, Hsu A, Yenokyan G, Jelin E. Pediatric surgeon opioid prescribing behavior: a survey of the American Pediatric Surgery Association membership. J Pediatr Surg. 2021;56(5):875-882. doi:10.1016/j.jpedsurg.2020.08.022 33039104

[17] Denning NL, Kvasnovsky C, Golden JM, Rich BS, Lipskar AM. Inconsistency in opioid prescribing practices after pediatric ambulatory hernia surgery. J Surg Res. 2019;241:57-62. doi:10.1016/j.jss.2019.03.043 31009886

[18] Van Cleve WC, Grigg EB. Variability in opioid prescribing for children undergoing ambulatory surgery in the United States. J Clin Anesth. 2017;41:16-20. doi:10.1016/j.jclinane.2017.05.014 28802595

[19] Groenewald CB. Opioid-prescribing patterns for pediatric patients in the United States. Clin J Pain. 2019;35(6):515-520. doi:10.1097/AJP.0000000000000707 30985396 PMC6782052

[20] Renny MH, Yin HS, Jent V, Hadland SE, Cerdá M. Temporal trends in opioid prescribing practices in children, adolescents, and younger adults in the US from 2006 to 2018. JAMA Pediatr. 2021;175(10):1043-1052. doi:10.1001/jamapediatrics.2021.1832 34180978 PMC8240008

[21] Chua KP, Brummett CM, Conti RM, Bohnert AS. Opioid prescribing to US children and young adults in 2019. Pediatrics. 2021;148(3):e2021051539. doi:10.1542/peds.2021-051539 34400571 PMC8778996

[22] McLaughlin C, Squillaro AI, Ourshaliman S, . The association between opioid use and outcomes in infants undergoing pyloromyotomy. Clin Ther. 2019;41(9):1690-1700. doi:10.1016/j.clinthera.2019.07.002 31409555 PMC7313351

[23] Squillaro AI, Ourshalimian S, McLaughlin CM, . Postoperative opioid analgesia impacts resource utilization in infants undergoing pyloromyotomy. J Surg Res. 2020;255:594-601. doi:10.1016/j.jss.2020.05.077 32652313 PMC7541571

[24] Corr TE, Hollenbeak CS. The economic burden of neonatal abstinence syndrome in the United States. Addiction. 2017;112(9):1590-1599. doi:10.1111/add.13842 28612362

[25] Percy Z, Brokamp C, McAllister JM, Ryan P, Wexelblatt SL, Hall ES. Subclinical and overt newborn opioid exposure: prevalence and first-year healthcare utilization. J Pediatr. 2020;222:52-58.e1. doi:10.1016/j.jpeds.2020.03.052 32423682 PMC7412356

[26] Apfeld JC, Kastenberg ZJ, Gibbons AT, Phibbs CS, Lee HC, Sylvester KG. The disproportionate cost of operation and congenital anomalies in infancy. Surgery. 2019;165(6):1234-1242. doi:10.1016/j.surg.2018.12.022 31056199

[27] Benneyworth BD, Bennett WE, Carroll AE. Cross-sectional comparison of critically ill pediatric patients across hospitals with various levels of pediatric care. BMC Res Notes. 2015;8:693. doi:10.1186/s13104-015-1550-9 26584713 PMC4653873

[28] Feudtner C, Feinstein JA, Zhong W, Hall M, Dai D. Pediatric complex chronic conditions classification system version 2: updated for ICD-10 and complex medical technology dependence and transplantation. BMC Pediatr. 2014;14:199. doi:10.1186/1471-2431-14-199 25102958 PMC4134331

[29] Ehwerhemuepha L, Donaldson CD, Kain ZN, . Race, ethnicity, and insurance: the association with opioid use in a pediatric hospital setting. J Racial Ethn Health Disparities. 2021;8(5):1232-1241. doi:10.1007/s40615-020-00882-9 33000430 PMC8552771

[30] Stake CE, Manworren RCB, Rizeq YK, Minhas S, Quan H, Barsness KA. Use of opioids and nonopioid analgesics to treat pediatric postoperative pain in the emergency department. Pediatr Emerg Care. 2022;38(1):e234-e239. doi:10.1097/PEC.0000000000002227 32941362

[31] Miller MM, Williams AE, Zapolski TCB, Rand KL, Hirsh AT. Assessment and treatment recommendations for pediatric pain: the influence of patient race, patient gender, and provider pain-related attitudes. J Pain. 2020;21(1-2):225-237. doi:10.1016/j.jpain.2019.07.002 31362065 PMC7960641

[32] Marin JR, Rodean J, Hall M, . Racial and ethnic differences in emergency department diagnostic imaging at US children’s hospitals, 2016-2019. JAMA Netw Open. 2021;4(1):e2033710. doi:10.1001/jamanetworkopen.2020.33710 33512517 PMC7846940

[33] Borrelli KN, Yao EJ, Yen WW, . Sex differences in behavioral and brainstem transcriptomic neuroadaptations following neonatal opioid exposure in outbred mice. eNeuro. 2021;8(5):ENEURO.0143-21.2021. doi:10.1523/ENEURO.0143-21.2021 34479978 PMC8454922

[34] Yen E, Kaneko-Tarui T, Ruthazer R, Harvey-Wilkes K, Hassaneen M, Maron JL. Sex-dependent gene expression in infants with neonatal opioid withdrawal syndrome. J Pediatr. 2019;214:60-65.e2. doi:10.1016/j.jpeds.2019.07.032 31474426 PMC10564583

[35] Zaveri S, Nobel TB, Khetan P, Srinivasan M, Divino CM. Surgeon bias in postoperative opioid prescribing. World J Surg. 2022;46(7):1660-1666. doi:10.1007/s00268-022-06532-x 35394230

[36] Schulson LB, Dick A, Sheng F, Stein BD. An exploratory analysis of differential prescribing of high-risk opioids by insurance type among patients seen by the same clinician. J Gen Intern Med. 2023;38(7):1681-1688. doi:10.1007/s11606-023-08025-6 36745303 PMC10212884

[37] O’Connell AA, Goldstein J, Rogers HJ, Peng CYJ. Multilevel logistic models for dichotomous and ordinal data. In: O’Connell AA, McCoach DB, eds. Multilevel Modeling of Educational Data. Information Age Publishing; 2008:199-242.

[38] Bryk AS, Raudenbush SW. Hierarchical Linear Models: Applications and Data Analysis Methods. Sage; 1992.

[39] Young LW, Hu Z, Annett RD, ; Eunice Kennedy Shriver National Institute of Child Health and Human Development Neonatal Research Network and the NIH Environmental Influences on Child Health Outcomes (ECHO) Program Institutional Development Awards States Pediatric Clinical Trials Network. Site-level variation in the characteristics and care of infants with neonatal opioid withdrawal. Pediatrics. 2021;147(1):e2020008839. doi:10.1542/peds.2020-008839 33386337 PMC7780957

[40] Liu G, Kong L, Leslie DL, Corr TE. A longitudinal healthcare use profile of children with a history of neonatal abstinence syndrome. J Pediatr. 2019;204:111-117.e1. doi:10.1016/j.jpeds.2018.08.032 30270164

[41] Patrick SW, Kaplan HC, Passarella M, Davis MM, Lorch SA. Variation in treatment of neonatal abstinence syndrome in US children’s hospitals, 2004-2011. J Perinatol. 2014;34(11):867-872. doi:10.1038/jp.2014.114 24921412

[42] Milliren CE, Gupta M, Graham DA, Melvin P, Jorina M, Ozonoff A. Hospital variation in neonatal abstinence syndrome incidence, treatment modalities, resource use, and costs across pediatric hospitals in the United States, 2013 to 2016. Hosp Pediatr. 2018;8(1):15-20. doi:10.1542/hpeds.2017-0077 29263122

[43] Gaddis N, Mathur R, Marks J, . Multi-trait genome-wide association study of opioid addiction: *OPRM1* and beyond. Sci Rep. 2022;12(1):16873. doi:10.1038/s41598-022-21003-y 36207451 PMC9546890

[44] Ren ZY, Xu XQ, Bao YP, . The impact of genetic variation on sensitivity to opioid analgesics in patients with postoperative pain: a systematic review and meta-analysis. Pain Physician. 2015;18(2):131-152. 25794200

[45] Balyan R, Zhang X, Chidambaran V, . OCT1 genetic variants are associated with postoperative morphine-related adverse effects in children. Pharmacogenomics. 2017;18(7):621-629. doi:10.2217/pgs-2017-0002 28470102 PMC5591462

[46] Tzvetkov MV. OCT1 pharmacogenetics in pain management: is a clinical application within reach? Pharmacogenomics. 2017;18(16):1515-1523. doi:10.2217/pgs-2017-0095 29061087

[47] Elens L, Norman E, Matic M, Rane A, Fellman V, van Schaik RH. Genetic predisposition to poor opioid response in preterm infants: impact of *KCNJ6* and *COMT* polymorphisms on pain relief after endotracheal intubation. Ther Drug Monit. 2016;38(4):525-533. doi:10.1097/FTD.0000000000000301 27027462

[48] Zimmerman KO, Smith PB, Benjamin DK, . Sedation, analgesia, and paralysis during mechanical ventilation of premature infants. J Pediatr. 2017;180:99-104.e1. doi:10.1016/j.jpeds.2016.07.001 27522446 PMC5183489

[49] Ten Barge JA, Vermeulen MJ, Simons SHP, van den Bosch GE. Pain management for necrotizing enterocolitis: getting the balance right. Pediatr Res. 2022;92(5):1423-1431. doi:10.1038/s41390-022-01968-2 35169278 PMC9700516

[50] Schneider JB, Sweberg T, Asaro LA, ; Randomized Evaluation of Sedation Titration for Respiratory Failure (RESTORE) Study Investigators. Sedation management in children supported on extracorporeal membrane oxygenation for acute respiratory failure. Crit Care Med. 2017;45(10):e1001-e1010. doi:10.1097/CCM.0000000000002540 28614197 PMC5600656

[51] Yalcin N, Sürmelioğlu N, Allegaert K. Population pharmacokinetics in critically ill neonates and infants undergoing extracorporeal membrane oxygenation: a literature review. BMJ Paediatr Open. 2022;6(1):e001512. doi:10.1136/bmjpo-2022-001512 36437518 PMC9639121

[52] Van Driest SL, Shah A, Marshall MD, . Opioid use after cardiac surgery in children with Down syndrome. Pediatr Crit Care Med. 2013;14(9):862-868. doi:10.1097/PCC.0b013e31829f5d9d 23962833 PMC3830692

[53] Patt E, Singhania A, Roberts AE, Morton SU. The genetics of neurodevelopment in congenital heart disease. Can J Cardiol. 2023;39(2):97-114. doi:10.1016/j.cjca.2022.09.026 36183910

[54] Nattel SN, Adrianzen L, Kessler EC, . Congenital heart disease and neurodevelopment: clinical manifestations, genetics, mechanisms, and implications. Can J Cardiol. 2017;33(12):1543-1555. doi:10.1016/j.cjca.2017.09.020 29173597

[55] McPherson C, Grunau RE. Neonatal pain control and neurologic effects of anesthetics and sedatives in preterm infants. Clin Perinatol. 2014;41(1):209-227. doi:10.1016/j.clp.2013.10.002 24524456 PMC3925313

[56] Ancora G, Lago P, Garetti E, . Evidence-based clinical guidelines on analgesia and sedation in newborn infants undergoing assisted ventilation and endotracheal intubation. Acta Paediatr. 2019;108(2):208-217. doi:10.1111/apa.14606 30290021

[57] Horton JD, Munawar S, Corrigan C, White D, Cina RA. Inconsistent and excessive opioid prescribing after common pediatric surgical operations. J Pediatr Surg. 2019;54(7):1427-1431. doi:10.1016/j.jpedsurg.2018.07.002 30057208

[58] Kelley-Quon LI, Kirkpatrick MG, Ricca RL, . Guidelines for opioid prescribing in children and adolescents after surgery: an expert panel opinion. JAMA Surg. 2021;156(1):76-90. doi:10.1001/jamasurg.2020.5045 33175130 PMC8995055

[59] Grabski DF, Vavolizza RD, Lepore S, . A quality improvement intervention to reduce postoperative opiate use in neonates. Pediatrics. 2020;146(6):e20193861. doi:10.1542/peds.2019-3861 33184168 PMC7706109

[60] Kelley-Quon LI, Ourshalimian S, Lee J, . Multi-institutional quality improvement project to minimize opioid prescribing in children after appendectomy using NSQIP-Pediatric. J Am Coll Surg. 2022;234(3):290-298. doi:10.1097/XCS.0000000000000056 35213491 PMC11559361

[61] Chiem JL, Franz A, Bishop N, Liston D, Low DK. An opioid sparing anesthesia protocol for pediatric open inguinal hernia repair: a quality improvement project. Pediatr Qual Saf. 2022;7(2):e548. doi:10.1097/pq9.0000000000000548 35369423 PMC8970091

[62] Jung JA, Higgins K, Lange P, Noda C. Evaluation of postoperative pain management using an opioid-sparing protocol in pediatric surgical patients. Am Surg. 2023;89(4):1024-1028. doi:10.1177/00031348211054709 34794319

[63] DiFiore JW, Robertson JO, Chhabada S, . Next day discharge after the Nuss procedure using intercostal nerve cryoablation, intercostal nerve blocks, and a perioperative ERAS pain protocol. J Pediatr Surg. 2022;57(2):213-218. doi:10.1016/j.jpedsurg.2021.10.034 34823843

