# Supplementary material for: Institutional and Regional Variation in Opioid Prescribing for Hospitalized Infants in the US
Source: JAMA Netw Open. 2024 Mar 12;7(3):e240555. doi: 10.1001/jamanetworkopen.2024.0555 (PMC10936113; doi:10.1001/jamanetworkopen.2024.0555)
Supplement: Supplement 1. — eTable 1. ICD-10 Codes Used to Build Study Cohort eTable 2. Opioid and Methadone Exposure in Premature vs Term Infants and Stratified by U.S. Region eTable 3. Cumulative Days of Opioid and Methadone Exposure in Study Cohort and Stratified by U.S. Region eTable 4. Cumulative Days of Opioid and Methadone Exposure in Premature vs Term Infants and Stratified by U.S. Region eFigure. Study Flow Diagram [file jamanetwopen-e240555-s001.pdf]

Supplementary Online Content

Keane OA, Ourshalimian S, Lakshmanan A, et al. Institutional and region variation in opioid prescribing for hospitalized infants in the US. *JAMA Netw Open*. 2024;7(3):e240555. doi:10.1001/jamanetworkopen.2024.0555

eTable 1. ICD-10 Codes Used to Build Study Cohort

eTable 2. Opioid and Methadone Exposure in Premature vs Term Infants and Stratified by U.S. Region

eTable 3. Cumulative Days of Opioid and Methadone Exposure in Study Cohort and Stratified by U.S. Region

eTable 4. Cumulative Days of Opioid and Methadone Exposure in Premature vs Term Infants and Stratified by U.S. Region

eFigure. Study Flow Diagram

This supplementary material has been provided by the authors to give readers additional information about their work.

© 2024 Kean OA et al. *JAMA Network Open.*

eTable 1. ICD-10 Codes Used to Build Study Cohort

High-Risk Infant Category	ICD-10 Codes
Congenital heart disease procedure (CHD Px)	021K4JP, 021K4JQ, 021K4JR, 021K4KP, 021K4KQ, 021K4KR, 021K4ZP, 021K4ZQ, 021K4ZR, 021L0ZW, 021L4ZW, 021K0KQ, 021K0KR, 021K0ZP, 021K0ZQ, 021K0ZR, 021K49P, 021K49Q, 021K49R, 021K4AP, 021K4AQ, 021K4AR, 02UW07Z, 02UW08Z, 02UW0JZ, 02UW0KZ, 02UW47Z, 02UW48Z, 02UW4JZ, 02UW4KZ, 025P0ZZ, 025P4ZZ, 025V0ZZ, 025V4ZZ, 025W0ZZ, 025W4ZZ, 02BP0ZZ, 02BP4ZZ, 02BQ0ZZ, 02BQ4ZZ, 02BR0ZZ, 02BR4ZZ, 02BS0ZZ, 02BS4ZZ, 02BT0ZZ, 02BT4ZZ, 02BV0ZZ, 02BV4ZZ, 02BW0ZZ, 02BW4ZZ, 02LR0CT, 02LR0DT, 02LR0ZT, 02LR4CT, 02LR4DT, 02LR4ZT, 02RP07Z 02RP08Z, 02RP0JZ, 02RP0KZ, 02RP47Z, 02RP48Z, 02RP4JZ, 02RP4KZ, 02RQ07Z, 02RQ08Z, 02RQ0JZ, 02RQ0KZ, 02RQ47Z, 02RQ48Z, 02RQ4JZ, 02RQ4KZ, 02RR07Z, 02RR08Z, 02RR0JZ, 02RR0KZ, 02RR47Z, 02RR48Z, 02RR4JZ, 02RR4KZ, 02RS07Z, 02RS08Z, 02RS0JZ, 2RS0KZ, 02RS47Z, 02RS48Z, 02RS4JZ, 02RS4KZ, 02RT07Z, 02RT08Z, 02RT0JZ, 02RT0KZ, 02RT47Z, 02RT48Z, 02RT4JZ, 02RT4KZ, 02RV07Z, 02RV08Z, 02RV0JZ, 02RV0KZ, 02RV47Z, 02RV48Z, 02RV4JZ, 02RV4KZ, 02RW07Z, 02RW08Z, 02RW0JZ, 02RW0KZ, 02RW47Z, 02RW48Z, 02RW4JZ, 02RW4KZ, 02SP0ZZ, 02SQ0ZZ, 02SR0ZZ, 02SS0ZZ, 02ST0ZZ 02SV0ZZ, 02SW0ZZ, 02UP07Z, 02UP08Z, 02UP0JZ, 02UP0KZ, 02UP47Z, 02UP48Z, 02UP4JZ, 02UP4KZ, 02UQ07Z, 02UQ08Z, 02UQ0JZ, 02UQ0KZ, 02UQ47Z, 02UQ48Z, 02UQ4JZ, 02UQ4KZ, 02UR07Z, 02UR08Z, 02UR0JZ, 02UR0KZ, 02UR47Z, 02UR48Z, 02UR4JZ, 02UR4KZ, 02US07Z 02US08Z, 02US0JZ, 02US0KZ, 02US47Z, 02US48Z, 02US4JZ, 02US4KZ, 02UT07Z, 02UT08Z, 02UT0JZ, 02UT0KZ ,02UT47Z, 02UT48Z, 02UT4JZ, 02UT4KZ, 02UV07Z, 02UV08Z, 02UV0JZ, 02UV0KZ, 02UV47Z, 02UV48Z, 02WM0JZ, 02WM4JZ, 02YA0Z0, 02YA0Z1, 02YA0Z2, 02UM07Z, 02UM0JZ, 02UM0KZ, 02UM47Z, 02UM48Z, 02UM4JZ, 02UM4KZ, 02VR0ZT, 02W50JZ, 02W54JZ, 02TM0ZZ, 02TM4ZZ, 02U507Z, 02U508Z, 02U50JZ, 02U50KZ, 02U547Z, 02U548Z, 02U54JZ, 02U54KZ, 02SP0ZZ, 02SW0ZZ, 02T50ZZ, 02T54ZZ, 02RG47Z, 02RG48Z, 02RG4JZ, 02RG4KZ, 02RH07Z, 02RH08Z, 02RH0JZ, 02RH0KZ, 02RH47Z, 02RH48Z, 02RH4JZ, 02RH4KZ, 02RJ07Z, 02RJ08Z, 02RJ0JZ, 02RJ0KZ, 02RJ47Z ,02RJ48Z, 02RJ4JZ, 02RJ4KZ, 02RF07Z, 02RF08Z, 02RF0JZ, 02RF0KZ, 02RF47Z, 02RF48Z, 02RF4JZ, 02RF4KZ, 02RG07Z, 02RG08Z, 02RG0JZ, 02RG0KZ, 02QF0ZZ, 02QF4ZZ, 02QG0ZZ, 02QG4ZZ, 02QH0ZZ, 02QH4ZZ, 02QJ0ZZ, 02QJ4ZZ, 02QM0ZZ, 02QM4ZZ ,02LR0ZT, 02550ZZ, 02570ZK, 02570ZZ, 02574ZK, 021K09P, 021K09Q, 021K09R, 021K0AP, 021K0AQ, 021K0AR, 021K0JP, 021K0JQ, 021K0JR, 021K0KP, 021609P, 021609Q, 021609R, 02160AP, 02160AQ, 02160AR, 02160JP, 02160JQ, 02160JR, 02160KP, 02160KQ, 02160KR, 02160ZP, 02160ZQ, 02160ZR, 021649P, 021649Q, 021649R, 02164AP, 02164AQ, 02164AR, 02164JP, 02164JQ, 02164JR, 02164KP, 02164KQ, 02164KR, 02164ZP, 02164ZQ, 02164ZR, 021709P, 021709Q, 021709R, 02170AP, 02170AQ, 02170AR, 02170JP, 02170JQ, 02170JR, 02170KP, 02170KQ, 02170KR, 02170ZP, 02170ZQ, 02170ZR, 021749P, 021749Q, 021749R, 02174AP, 02174AQ, 02174AR, 02174JP, 02174JQ, 02174JR, 02174KP, 02174KQ, 02174KR, 02174ZP, 02174ZQ, 02174ZR, 02LR3DT, 02LR3ZT, 02LR3CT, 02LR4DT, 02Q50ZZ, 02PY3DZ, 02U53JZ, 02VP0CZ, 027S3ZZ, 02C60ZZ, 02PA3DZ, 03HP33Z, 027T34Z, 02B50ZZ, 02PY0CZ, 02PY0DZ, 02UM08Z, 02713FZ, 027H3ZZ, 027Q3ZZ, 027R3DT, 027R3DZ, 027R3ZZ, 027T3ZZ, 028D0ZZ, 02B60ZZ, 02BJ0ZZ, 02C70ZZ, 02CQ0ZZ, 02CW0ZZ, 02HL0MZ, 02HN3JZ, 02HP3DZ, 02HQ3DZ, 02HV3DZ, 02JA0ZZ,

© 2024 Kean OA et al. *JAMA Network Open.*

	02Q53ZZ, 02Q60ZZ, 02QQ0ZZ, 02QX0ZZ, 02U508Z, 02U50JZ, 02UJ07G, 02UP0JZ, 02UR07Z, 02UW3JZ, 02VQ0CZ, 02VR3CT
Extracorporeal Membrane Oxygenation (ECMO)	Z92.81, 5A15223, 5A1522G
Hypoxic Ischemic Encephalopathy (HIE)	P91.60, P91.61, P91.62, P91.63
Medical Necrotizing Enterocolitis (NEC)	P77.1, P77.2, P77.3, P77.9, K55.30, K55.31, K55.32, K55.33
Surgical Necrotizing Enterocolitis (NEC Px)	*One of the codes for NEC plus one of the following procedural codes: 0DB80ZX, 0DB80ZZ, 0DB84ZX, 0DB84ZZ, 0DB90ZX, 0DB90ZZ, 0DB94ZX, 0DB94ZZ, 0DBA0ZX, 0DBA0ZZ, 0DBA4ZX, 0DBA4ZZ, 0DBB0ZX, 0DBB0ZZ, 0DBB4ZX, 0DBB4ZZ, 0DT80ZZ, 0DT84ZZ, 0DT90ZZ, 0DT94ZZ, 0DTA0ZZ, 0DTA4ZZ, 0DTB0ZZ, 0DTB4ZZ, 0D9W00Z, 0D9W0ZX, 0D9W0ZZ, 0D9W40Z, 0D9W4ZX, 0D9W4ZZ, 0DJW0ZZ, 0DJW4ZZ, 0DBE0ZX, 0DBE0ZZ, 0DBE4ZX, 0DBE4ZZ, 0DBF0ZX, 0DBF0ZZ, 0DBF4ZX, 0DBF4ZZ, 0DBG0ZX, 0DBG0ZZ, 0DBG4ZX, 0DBG4ZZ, 0DBGFZZ, 0DBH0ZX, 0DBH0ZZ, 0DBH4ZX, 0DBH4ZZ, 0DBK0ZX, 0DBK0ZZ, 0DBK4ZX, 0DBK4ZZ, 0DBL0ZX, 0DBL0ZZ, 0DBL4ZX, 0DBL4ZZ, 0DBLFZZ, 0DBM0ZX, 0DBM0ZZ, 0DBM4ZX, 0DBM4ZZ, 0DBMFZZ, 0DBN0ZX, 0DBN0ZZ, 0DBN4ZX, 0DBN4ZZ, 0DBNFZZ, 0DTN0ZZ, 0DTN4ZZ, 0DTM0ZZ, 0DTM4ZZ, 0DTH0ZZ, 0DTH4ZZ, 0DTK0ZZ, 0DTK4ZZ, 0DTL0ZZ, 0DTL4ZZ
Abdominal Surgery	Z93.1, Q44.2, K74.4, K74.5, K74.6, Q79.3, Q79.2, Q43.3, K56.2, Q43.1, K44.9, Q39.0, Q39.1, Q39.2, Q39.3, Q39.4, Q41, Q41.0. Q41.1, Q41.2, Q41.8, Q41.9, K21.0, K44.9, Q79.0, Q79.1, Q40.1, Q89.9, P78.0, K63.1, P78.1, K65.9, K65.0, K65.8, K65.1, K66.1, K27.9, P78.82, K25.1, K25.9, K25.3, Q45.1, K55.0, K55.01, K55.011, K55.012, K55.019, K55.02, K55.021, K55.022, K55.029, K55.03, K55.031, K55.032, K55.039, K55.04, K55.041, K55.042, K55.049, K55.05, K55.051, K55.052, K55.059, K55.06, K55.061, K55.062, K55.069, K55.1, K55.8, K55.9, P76.0, P76.9, P76.8, K56.5, K56.50, K56.51, K56.52, K56.6, K56.60, K56.600, K56.601, K56.609, K56.69, K56.690, K56.691, K56.699, K22.2, K91.3, K91.30, K91.31, K91.32, Z93.2, Z43.3, Z98.0, T79.A3, T79.A3XA, T79.A3XD, T79.A3XS, Z90.49, Q41.8, Z90.49, Q41.8, Z90.49, Q41.8, K63.89, K91.89, K90.00, K90.01, K90.02, K90.03, K94.09, K94.10, K94.11, K94.12, K94.13, K94.19, K93.4, K94.19, Z53.31
Very Low Birthweight (VLBW)	P05.04, P05.14, P07.14, P05.06, P05.16, P07.16
Extremely Low Birthweight (ELBW)	P05.01, P05.11, P07.00, P07.01, P05.02, P05.12, P07.02, P05.03, P05.13, P07.03, P05.05, P05.15

© 2024 Kean OA et al. *JAMA Network Open.*

eTable 2. Opioid and Methadone Exposure in Premature vs Term Infants and Stratified by U.S. Region

	Total Cohort		Midwest		Northeast		South		West	
**Exposure (Y/N)**	**N**	**%**	**N**	**%**	**N**	**%**	**N**	**%**	**N**	**%**
**Term Infants (n=92484)**										
Any opioids, includes methadone	76043	82.2	18917	77.5	10959	80.5	29697	84.1	16470	86
Fentanyl	66769	72.2	16739	68.6	8652	63.6	27054	76.6	14324	74.8
Morphine	63513	68.7	15476	63.4	9499	69.8	24260	68.7	14278	74.6
Hydromorphone	6947	7.5	1509	6.2	679	5	2571	7.3	2188	11.4
Opioids, excludes methadone	76043	82.2	18917	77.5	10959	80.5	29697	84.1	16470	86
Methadone	8721	9.4	2028	8.3	740	5.4	4415	12.5	1538	8
**Preterm Infants (n=40174)**										
Any opioids, includes methadone	25428	63.3	8019	60.6	2426	54.8	10699	66.5	4284	66.6
Fentanyl	21484	53.5	6734	50.9	2160	48.8	9201	57.2	3389	52.7
Morphine	16941	42.2	5543	41.9	1584	35.8	6501	40.4	3313	51.5
Hydromorphone	778	1.9	146	1.1	135	3.0	245	1.5	252	3.9
Opioids, excludes methadone	25428	63.3	8019	60.6	2426	54.8	10699	66.5	4284	66.6
Methadone	1705	4.2	466	3.5	97	2.2	863	5.4	279	4.3

© 2024 Kean OA et al. *JAMA Network Open.*

eTable 3. Cumulative Days of Opioid and Methadone Exposure in Study Cohort and Stratified by U.S. Region

	Total Cohort		Midwest		Northeast		South		West		P-value
**Cumulative Days of Use**	**Median**	**IQR**	**Median**	**IQR**	**Median**	**IQR**	**Median**	**IQR**	**Median**	**IQR**	
Any opioids, includes methadone	5	2-12	5	2-13	5	3-12	5	2-12	5	2-11	<.001
Fentanyl	2	1-5	2	1-4	2	1-6	2	1-5	2	1-4	<.001
Morphine	4	2-8	4	2-11	4	2-9	4	2-8	3	2-7	<.001
Hydromorphone	4	1-14	2	1-9	7	2-26	3	1-11	5	1-17	<.001
Opioids, excludes methadone	5	2-11	5	2-12	5	2-11	5	2-11	5	2-11	<.001
Methadone	19	7-46	21	7-53	23	11-54	18	7-43	16	6-43	<.001

© 2024 Kean OA et al. *JAMA Network Open.*

eTable 4. Cumulative Days of Opioid and Methadone Exposure in Premature vs Term Infants and Stratified by U.S. Region

	**Total Cohort**		**Midwest**		**Northeast**		**South**		**West**		**P-value**
**Cumulative Days of Use**	**N**	**%**	**N**	**%**	**N**	**%**	**N**	**%**	**N**	**%**	
**Term Infants (n=92484)**											
Any opioids, includes methadone	5	3-11	5	2-11	5	3-11	5	3-12	5	3-10	<.001
Fentanyl	2	1-5	2	1-4	2	1-5	2	1-5	2	1-4	<.001
Morphine	4	2-7	4	2-9	4	2-8	4	2-7	3	2-7	<.001
Hydromorphone	3	1-13	2	1-8	6	2-24	2	1-10	5	1-16	<.001
Opioids, excludes methadone	5	3-11	5	2-11	5	3-11	5	3-11	5	3-10	<.001
Methadone	17	6-42	18	5-46	22	11-50.5	16	5-40	15	5-39	<.001
**Preterm Infants (n=40174)**											
Any opioids, includes methadone	4	1-15	4	1-17	5	2-19	4	1-13	4	1-16	<.001
Fentanyl	2	1-5	2	1-5	3	1-7	2	1-5	2	1-5	<.001
Morphine	5	2-17	5	2-19	5	2-22	4	1-14	4	1-15	<.001
Hydromorphone	8	2-28	6.5	1-20	12	5-35	8	1-24	8	1-28.5	0.001
Opioids, excludes methadone	4	1-15	4	1-17	5	2-19	3	1-12	4	1-15	<.001
Methadone	31	13-71	37	17-84	35	15-79	26	12-61	31	11-66	<.001

© 2024 Kean OA et al. *JAMA Network Open.*

eFigure. Study Flow Diagram

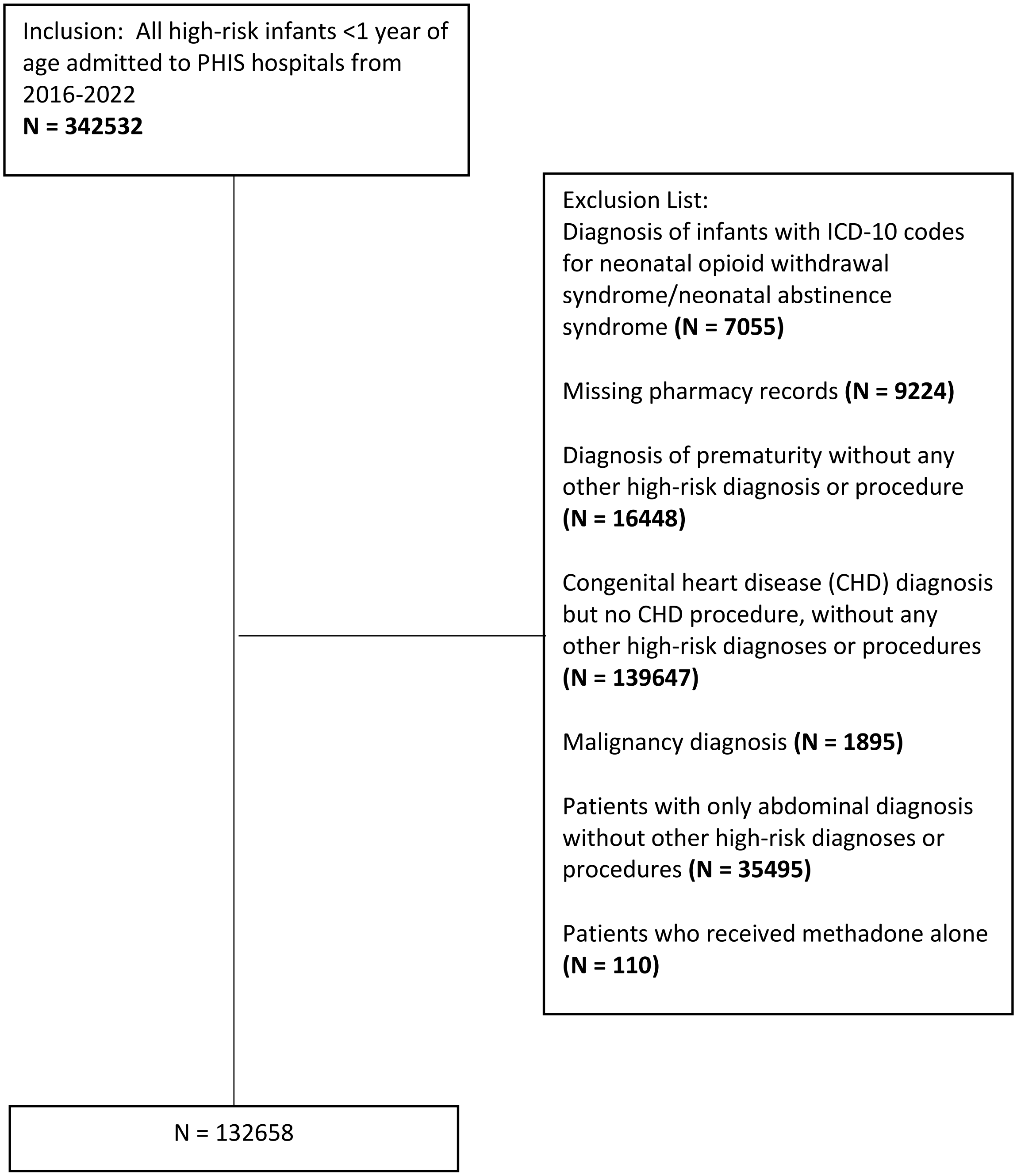

© 2024 Kean OA et al. *JAMA Network Open.*
