# Supplementary material for: Institutional and Regional Variation in Opioid Prescribing for Hospitalized Infants in the US
Source: JAMA Netw Open. 2024 Mar 12;7(3):e240555. doi: 10.1001/jamanetworkopen.2024.0555 (PMC10936113; doi:10.1001/jamanetworkopen.2024.0555)
Supplement: Supplement 2. — Data Sharing Statement [file jamanetwopen-e240555-s002.pdf]

Data Sharing Statement

Keane. Institutional and Regional Variation in Opioid Prescribing for Hospitalized Infants in the US. *JAMA Netw Open*. Published March 12, 2024. doi:10.1001/jamanetworkopen.2024.0555

Data
Data available: Yes
Data types: Deidentified participant data
How to access data: requests can be made to lkquon@chla.usc.edu
When available: With publication

Supporting Documents
Document types: Statistical/analytic code
How to access documents: requests can be made to lkquon@chla.usc.edu
When available: With publication

Additional Information
Who can access the data: Researchers whose proposed use of data has been approved
Types of analyses: For analysis that progresses the field further
Mechanisms of data availability: After approval of a proposal
